# People with recurrent ankle sprains do not change their ankle strategy in anticipation of a perturbation event

**DOI:** 10.1186/1757-1146-7-S1-A33

**Published:** 2014-04-08

**Authors:** Claire E Hiller, Stuart Blair, Elizabeth J Nightingale, Milena Simic, Joshua Burns

**Affiliations:** 1 Arthritis & Musculoskeletal Research Group, Faculty of Health Sciences, University of Sydney, NSW, Australia; 2 Institute for Neuroscience and Muscular Research, The Children's Hospital at Westmead, Sydney, NSW, Australia

## Background

It is unclear why people with an ankle sprain continue to resprain. People that do resprain take longer to return to equilibrium after an ankle perturbation event suggesting there is a change in the sensorimotor system [1,2].

## Aim

The aim of this study was to determine whether anticipation of an ankle inversion perturbation changes the ankle strategy used by participants with an inversion perturbation.

## Methods

Two groups of participants were recruited: 14 with no history of ankle injury (age 22.6 ± 0.7 yrs, 10 females) and 14 with a history of two or more ankle sprains (Age 21.1 ± 0.2 yrs, 9 females, 6 ± 2.5 sprains). Participants stood in single leg stance on an inversion perturbation platform. The perturbation platform dropped 15 degrees in the frontal plane on a trigger activated by the researcher. Movement Oscillation at the ankle was measured via a 3Space fastrak (Polhemius Ltd) with a receiver taped one cm above the lateral malleolus. Oscillation was determined as the standard deviation of the movement in the frontal plane (mm) measured over 10s while the platform was horizontal. Three conditions were investigated: standing with no change in the platform, standing with a 15 deg drop occurring at a specified time, and standing with a drop occurring at anytime. Data were compared between groups using a Mann-Whitney U test, as the data were not normally distributed.

## Results

There was a significant difference between groups for the no change condition with the control group holding their ankle within a tighter oscillation range than the injured group (Table 1). There was no difference between the groups for the other two conditions.

**Table 1:** Median and Interquartile range of the standard deviation of ankle oscillation in the frontal plane.

Platform condition	Instability group	Control Group	p*
No perturbation (mm)	0.085 (0.09)	0.054 (0.03)	0.023*

Perturbation at Specific time (mm)	0.090 (0.09)	0.067 (0.06)	0.646

Perturbation Anytime (mm)	0.088 (0.10)	0.074 (0.03)	0.581

## Discussion

The uninjured group increased the range of ankle oscillation in the frontal plane when an inversion drop was anticipated, which implies they are able to change their envelop of stability to meet changing conditions. The participants who had recurrent sprains had one strategy during single leg stance and were not able to change their oscillation across the conditions. Previous research using a perturbation drop may have over-estimated the time it takes uninjured participants to reach equilibrium after a perturbation as the no change condition is used as the baseline rather than the oscillation immediately prior to a drop [2].

## Conclusions

Consideration of the baseline measure in perturbation tests should be further explored. The lack of ability to change the envelop of stability in anticipation of an ankle rolling event is worth investigating in people with recurrent ankle sprains.
